# Effects of *Yucca schidigera* extract on serum biochemical parameters, humoral immune response, and intestinal health in young pigeons

**DOI:** 10.3389/fvets.2022.1077555

**Published:** 2023-01-12

**Authors:** Xiaoshan Sun, Zheng Wang, Xing Li, Shaohua Du, Dongmei Lin, Yuxin Shao

**Affiliations:** ^1^Pigeon Breeding Laboratory, Institute of Animal Husbandry and Veterinary Medicine, Beijing Academy of Agriculture and Forestry Sciences, Beijing, China; ^2^College of Life Science and Food Engineering, Hebei University of Engineering, Handan, China

**Keywords:** *Yucca schidigera* extract, young pigeons, serum indexes, immune function, intestinal flora

## Abstract

**Introduction:**

It is of great importance to find antibiotic alternatives that can improve poultry performance and enhance immunity. Plant-derived extracts and their concentrates are natural bioactive compounds that are widely and effectively applied as the antibiotic alternatives in animal industries. This study was conducted to investigate the effects of *Yucca schidigera* extract (YSE) on growth performance, serum biochemical parameters, immune function, intestinal morphology, and microbiota diversity of young pigeons.

**Methods:**

A total of 120 healthy White King pigeons (28 days old) with similar weight were randomly assigned to 4 treatments with six replicate cages. Each of the pigeons from 4 treatments was orally administrated with 0 (control), 5, 10, and 15 mg YSE per day, respectively.

**Results:**

The results showed that orally supplemental YSE had no significant effects (*P* > 0.05) on the growth performance and immune organ index of pigeons. The serum total protein and IgM contents in the 10 mg YSE group were significantly higher (*P* < 0.05) than those in the control group. Supplemental 10 and 15 mg YSE significantly lowered the level of serum total cholesterol (*P* < 0.05) and increased (*P* < 0.05) the villi height in the jejunum compared with the control group. Supplemental 5 and 10 mg YSE significantly decreased (*P* < 0.05) the level of serum alanine aminotransferase and the crypt depth in the ileum compared with the control group. The beta diversity showed a distinct difference in the ileum microbial composition between the control and the 10 mg YES group. YSE supplementation enriched the bacterial genera *Sulfurospirillum, Solobacterium, Desulfovibrio, Desulfobulbus, Lactococcus, Parabacteroides, Acidaminococcus, Acetobacter*, and *Streptococcus*. Additionally, *Enterococcus* genus showed a significantly negative correlation with serum alanine aminotransferase (*R* = −0.618, *P* = 0.043). *Actinomyces* genus showed a significantly negative correlation with cholesterol (*R* = −0.633, *P* = 0.036). *Turicibacter* genus showed a significantly positive correlation with villi height in the jejunum (*R* = 0.751, *P* = 0.008).

**Discussion:**

In conclusion, orally supplemental YSE could improve serum biochemistry, immunoglobulin contents, and intestinal morphology by regulating the composition of microbial community in the ileum of young pigeons.

## Introduction

Currently, domestic pigeons (*Columba livia*) are raised as a sort of commercial meat-type poultry in China (1), with a yearly production of around 680 million squabs, accounting for 80% of worldwide production (2). However, pigeons are the reservoirs of *Salmonella* and *Trichomonas gallinae*, and young pigeons during 1–3 months old are very susceptible to infection because of the weaning stress (3, 4). Although antibiotics have played a critical role in preventing pathogen infection and promoting animal production, the food and drug security organizations have called for restricting the use of antibiotics and chemotherapies in poultry, livestock, and aquaculture because of their adverse consequence on the development of antimicrobial resistance, and thereby posing a potential threat to human health (5–7). Thus, it is urgent to find antibiotic alternatives to improve growth performance, enhance immunity, and reduce stress of young pigeons (8). Plant-derived extracts and their concentrates are natural bioactive compounds that are widely and effectively applied as the antibiotic alternatives in animal industries (9, 10). Our team previously conducted an *in vitro* study on the functions of eleven plant extracts, and finally found that *Yucca schidigera* extract (YSE) can effectively inhibit the number of *Escherichia coli, Salmonella* and *Trichomonas*, and promote the growth of *Lactobacillus*. Hence, we decided to further explore the potential role of YSE in young pigeons.

As a medicinal plant extract, YSE has been widely used as a natural additive in food industries. It has numerous health benefits, including hypocholesterolemic, hypoglycemic, anti-inflammatory, antioxidant, anticancer, and immunostimulatory properties (11). Besides, YSE application in aquatic animals could effectively improve feed utilization, growth rate, and immune responses (12). And, it has also been demonstrated that in the livestock and poultry industries, YSE plays a significant role in promoting growth and production performance and reducing ammonia emission (13, 14). It was reported that dietary supplemental YSE could improve intestinal barrier function and reduce nitrogen emission in sow and weaned piglets (15, 16). Furthermore, *Yucca schidigera* administration increased the digestion of essential minerals, improved intestinal health and reduced the growth of pathogenic bacteria in broilers (17–19). However, whether supplemental YSE has benefits on young pigeons' intestinal health and immune system function has not been examined. We hypothesized that adding an appropriate level of YSE could improve the intestinal health and immune function of young pigeons. The current study's objective was to assess the effects of supplemental YSE on growth performance, serum biochemical indices, immune system function, and intestinal health, which could provide a theoretical basis for determining the appropriate requirment of YSE for young pigeons.

## Materials and methods

### Animals and treatment procedure

The study was carried out in accordance with the guidelines set by the Animal Care and Use Committee (permit number: SYXK-2017-0005) of the Institute of Animal Husbandry and Veterinary Medicine, Beijing Academy of Agriculture and Forestry Sciences (IAHVM-BAAFS), Beijing, China. The protocols were approved by the Animal Care and Use Committee of IAHVM-BAAFS.

### Birds, diets, and experimental design

A total number of 120, 28-day-old healthy White King pigeons with similar weight and size were randomly divided into four treatments, each consisting of six replicate cages (five birds per cage). The four groups were the control group, 5 mg YSE addition group (YSE5), 10 mg YSE addition group (YSE10), and 15 mg YSE addition group (YSE15). The pigeons were fed a mixed grain diet, and the compound feed was composed of maize, pea, soybean meal, wheat, and sorghum. The feed, sand, and water were available *ad libitum*. The ingredients and nutrient levels are shown in Table 1. Birds were raised in a room under a 16 h light and 8 h darkness cycle, and the temperature was kept at 22 ± 6°C during the entire experimental period. After acclimatization for 1 week, the control group was orally administrated with 1mL/bird/day 0.9% NaCl solution, and the other groups were orally administrated with 1mL/bird/day YSE solution. All the birds were gavaged by a pipette with sterilized tips at 10 am once a day after feeding 3h throughout the entire experiment. The YSE was dissolved in 0.9% NaCl solution with the final concentration of 5, 10, and 15 mg/mL. We selected the tested levels of YSE according to the published studies about the roles of YSE in quails and hens (11, 20). Based on their dietary YSE levels (50, 100, and 150 mg/kg) and daily feed intakes (0.09 kg/d for hens), the levels of YSE (5, 10, and 15 mg/d per pigeon) were used in this study. The experiment lasted 14 days.

**Table 1:** Ingredient compositions and nutrient levels of basal diets for young pigeons (on as-fed basis)^a^.

**Ingredients**	**Content %**	**Nutrient composition^c^**	
Maize	37.60	Metabolizable energy (Kcal/kg)^c^	2,796
Wheat	12.30	Crude protein^d^	14.5%
Sorghum	10.80	Calcium^d^	1.1%
Pea	20.80	Phosphorus^d^	0.5%
Soybean meal	13.52		
Soybean oil	1.00		
CaHPO_4_·2H_2_O	2.20		
Limestone	1.00		
NaCl	0.40		
DL-Methionine	0.12		
Vitamin and mineral premix^b^	0.16		

a The diet was composed of 70% whole-grain (maize, pea, wheat, and sorghum) and 30% pellet feed, and the pellet feed was composed of crushed maize, soybean meal, soybean oil, CaHPO_4_·2H_2_O, limestone, NaCl, DL-Methionine and vitamin and mineral premix.

b Supplied per kilogram of diet:vitamin A 4000 IU, vitamin D3 1725 IU, vitamin E 20 U, vitamin K3 1.0 mg, vitamin B1 2.5 mg, vitamin B2 8 mg, vitamin B6 2.5 mg, vitamin B12 0.015 mg, Pantothenic acid 7.5 mg, Niacin 15 mg, Folic acid 0.55 mg, Biotin 0.12 mg, Choline 200 mg, Zn (ZnSO_4_.7H_2_O) 50 mg, Cu (CuSO4.5H_2_O) 8 mg, Mn (MnSO_4_.H_2_O) 55 mg, Fe (FeSO_4_•7H_2_O) 40 mg, I (KI) 0.35 mg, Se (Na_3_SeO_3_) 0.15 mg.

c Calculated values.

d Analyzed values and each value based on triplicate determinations.

### YSE product and the main ingredients

The YSE (20.0% purity) was purchased from a commercial supplier (Xi'an Ruidi Biotechnology Co., Ltd., Xi'an, China). It is in the form of brown yellow powder with good water solubility. The main ingredients of YSE include the total flavonoids, phenols, polysaccharides, and saponins, and they were determined by the biochemicals kits (Norminkoda Biotechnology Co., Ltd. Wuhan, China). The contents of total flavonoids, phenols, polysaccharides, and saponins were 8.02 ± 0.058, 8.22 ± 0.240, 566.5 ± 7.071, 27.3 ± 0.853 mg/g, respectively based on triplicate assays.

### Growth performance

Before weighing, the pigeons fasted for 12 h. Feed intake was recorded weekly. Pigeons, feed, and sand were weighed at 8 am on the first and last days of the experiment. Initial body weight (IBW), final body weight (FBW), average daily gain (ADG), average daily feed intake (ADFI), and the ratio of feed:gain (F/G) were measured and calculated at the end of the experiment.

### Sample collection

On the 14th day of the experiment, six pigeons from each treatment (one bird from each replicate) were randomly selected. And, 7 mL of blood was collected aseptically from the wing portal vein and centrifuged at 3,000 × g for 10 min at 4°C, and the serum was obtained and stored at −20°C for analysis of serum biochemical indexes. After collecting the blood, pigeons were killed by jugular exsanguination. The immune organs including the spleen, bursa, and thymus were collected and weighed after stripping fat, and the immune organ index was expressed as the organ weight relative to the living body weight (g/kg). Next, ~2-cm long segments of duodenum (the midpoint), jejunum (preceding the Meckel's diverticulum) and ileum (preceding the ileocecal junction) were separated and washed in PBS, fixed in 4% paraformaldehyde solution and kept at 4°C for histological morphology analysis. The ileal contents were collected aseptically and stored at −80°C for DNA extraction.

### Serum biochemical parameters and immunoglobulins contents

The levels of total superoxide dismutase (T-SOD), total antioxidant capacity (T-AOC), alanine aminotransferase (ALT), aspartate aminotransferase (AST), total triglyceride (TG), total cholesterol (T-CHO), albumin (ALB) and total protein (TP) in serum were determined by the commercial testing kits (Nanjing Jiancheng Bioengineering Institute, Nanjing, China). Serum immunoglobulins concentrations, including immunoglobulin A (IgA), immunoglobulin G (IgG), and immunoglobulin M (IgM) were determined by ELISA tests (Bird IgA/IgG/IgM ELISA Quantitation kits, Shanghai Enzyme-linked Biotechnology Co., Ltd. Shanghai, China) following the manufacturer's protocol.

### Intestinal histomorphology

Paraformaldehyde-fixed intestinal tissues were embedded in paraffin. Serial slices (5 μm) were prepared using haematoxylin and eosin staining and observed for histomorphology under an Olympus optical microscope using ProgRes CapturePro software, version 2.7 (Jenoptik, Germany). The villus height (VH) and crypt depth (CD) were measured from 10 randomly selected villi and associated crypts with one section per bird at 40 × magnification. The ratio of VH:CD was then calculated from the above measurements by dividing the villus height by the crypt depth.

### DNA extraction and PCR amplification

The ileal contents were collected in sterilized plastic tubes and then immediately stored in liquid nitrogen to investigate the intestinal microflora. Total bacterial DNA was extracted from the collected digest samples using an E.Z.N.A. Stool DNA Kit (Omega Bio-Tek, Norcross, GA, USA) according to the manufacturer's protocol. The final DNA concentration and purity were determined by a NanoDrop 2000 UV–Vis spectrophotometer (Thermo Scientific, Wilmington, USA), and DNA quality was checked by 1% agarose gel electrophoresis. The V3-V4 hypervariable regions of the bacterial 16S rRNA gene were amplified with primers 338F (5′-ACTCCTACGGGAGGCAGCAG-3′) and 806R (5′-GGACTACHVGGGTWTCTAAT-3′) (21, 22) using a thermocycler PCR system (GeneAmp 9700, ABI, Vernon, CA, USA). The PCR reactions were conducted with a high-fidelity polymerase using the following program: 3 min of denaturation at 95°C, 27 cycles of 30 s at 95°C, 30 s for annealing at 55°C, and 45 s for elongation at 72°C, and a final extension at 72°C for 10 min. PCR reactions were performed in triplicate according to previous studies (22, 23). The PCR products were visualized in a 2% agarose gel, and then purified using an AxyPrep DNA Gel Extraction Kit (Axygen Biosciences, Union City, CA, USA), and quantified using QuantiFluor-^TM^ST (Promega, USA) according to the manufacturer's protocol (23).

### Illumina MiSeq sequencing of 16S rRNA gene

Purified amplicons were pooled in equimolar amounts and paired-end sequenced (2 × 300) on an Illumina MiSeq platform (Illumina, San Diego, USA) according to the standard protocols (24) by Majorbio Bio-Pharm Technology Co., Ltd. (Shanghai, China). The raw reads were deposited into the NCBI Sequence Read Archive (SRA) database (accession number: PRJNA81814).

### Processing of sequencing data

Raw fastq files were quality filtered by Trimmomatic software and merged by FLASH software (version 1.2, https://ccb.jhu.edu/sofware/FLASH/index.shtml) (22, 23). Operational taxonomic units (OTUs) were clustered with a 97% similarity cutoff using UPARSE (version 7.1, https://www.drive5.com/uparse/) analysis (25). The taxonomy of each 16S rRNA gene sequence was analyzed by the Ribosomal Database Project Classifier algorithm (version 11.1) against the Silva (SSU128) 16S rRNA database using a confidence threshold of 70% (The result of species annotation has the highest reliability using 70% confidence threshold) (26).

### Statistical analyses

All data were analyzed using SPSS statistical software (version 20.0) and presented as means ± standard error. Differences were evaluated by one-way ANOVA, and comparisons between multiple groups were analyzed by *post-hoc* Duncan's multiple range test. Polynomial orthogonal contrasts were used to determine the linear and quadratic responses of dependent variables to YSE levels. The *P* < 0.05 were considered to be significant, and 0.05 ≤ *P* < 0.10 was defined as a tendency.

The beta diversity was analyzed by principal coordinate analysis diagram (PCoA) and hierarchical clustering by unweighted pair group method with arithmetic average (UPGMA). Kruskal-Wallis tests with Wilcoxon test were used to visualize the highly significant abundant taxa at phylum and genus levels among the groups.

Linear discriminant analysis for effect size (LEfSe) identifies features that are statistically different among biological classes (Kruskal-Wallis sum-rank test, *P* < 0.05). It then performs additional tests to assess whether these differences are consistent with respect to expected biological behavior (Wilcoxon ran-sum test, *P* < 0.05). Lastly, the effect relevance is estimated by the linear discriminant analysis (LDA) effect size. Pearson correlation coefficients between the top 20 abundant bacterial genera and serum biochemical indexes or intestinal morphology were displayed on the heat map.

## Results

### Growth performance

The data of growth performance were shown in Table 2. Supplementation of different levels of YSE had no significant effects (*P* > 0.05) on IBW, FBW, ADG, ADFI and F/G of the young pigeons.

**Table 2:** The effect of supplemental different levels of YSE on the growth performance of young pigeons^a^.

**Items**	**Control**	**YSE5**	**YSE10**	**YSE15**	***P*-value**	**Linear**	**Quadratic**
IBW(g)	433.7 ± 10.8	437.0 ± 3.61	436.9 ± 7.84	436.4 ± 5.46	0.998	0.974	0.843
FBW(g)	463.8 ± 35.4	461.4 ± 8.80	463.5 ± 22.9	469.5 ± 25.3	0.950	0.680	0.682
ADG(g/d)	2.15 ± 2.29	1.74 ± 0.80	1.90 ± 1.58	2.36 ± 1.64	0.920	0.797	0.527
ADFI(g/d)	41.6 ± 6.32	41.4 ± 4.36	41.4 ± 7.39	44.6 ± 4.44	0.730	0.415	0.473
F/G	16.55 ± 6.68	29.17 ± 14.75	23.75 ± 11.27	22.29 ± 17.16	0.554	0.682	0.265

a n = 6 replicates (5 young pigeons per replicate) per treatment.

Control, 0.9% NaCl solution; YSE5, 5 mg/mL YSE; YSE10, 10 mg/mL YSE; YSE15, 15 mg/mL YSE.

IBW, initial body weight; FBW, final body weight; ADG, average daily gain; ADFI, average daily feed intake; F/G, feed/ gain ratio.

### Immune organ index

The immune organ indexes, including spleen, bursa, and thymus indexes were shown in Table 3. No significant differences were observed in spleen and thymus indexes among the four groups (*P* > 0.05).

**Table 3:** Effects of supplemental different levels of YSE on immune organ index of young pigeons (g/kg)^a^.

**Items**	**Control**	**YSE5**	**YSE10**	**YSE15**	***P*-value**	**Linear**	**Quadratic**
Thymus index	2.00 ± 1.09	1.80 ± 0.78	1.90 ± 0.75	1.10 ± 0.91	0.343	0.150	0.469
Bursa of Fabricius index	1.80 ± 0.39	1.70 ± 0.48	1.20 ± 0.37	1.40 ± 0.22	0.090	0.280	0.431
Spleen index	1.20 ± 0.22	1.20 ± 0.31	1.40 ± 0.48	1.30 ± 0.71	0.910	0.644	0.855

a n = 6 replicates (5 young pigeons per replicate) per treatment.

Control, 0.9% NaCl solution; YSE5, 5 mg/mL YSE; YSE10, 10 mg/mL YSE; YSE15, 15 mg/mL YSE.

### Serum biochemical parameters and immunoglobulin contents

The comparison of serum biochemical parameters was shown in Table 4. The content of TP increased linearly and quadratically with the increase of YSE supplementation (*P* < 0.05), and pigeons gavaged with 10 mg YSE had a higher level of TP than the the control pigeons (*P* < 0.05). The level of T-CHO decreased significantly linearly with the increase of YSE addition (*P* < 0.05), and pigeons gavaged with 5 and 10 mg YSE had lower levels of T-CHO than the control pigeons (*P* < 0.05). The level of ALT decreased quadratically with the increase of YSE supplementation (*P* < 0.05), and compared with the control group, orally supplemental 10 and 15 mg YSE significantly decreased the levels of ALT (*P* < 0.05). There was no significant difference in the activities of T-SOD, T-AOC, ALB, TG, and AST among the four groups (*P* > 0.05).

**Table 4:** Effects of supplemental different levels of YSE on serum biochemical parameters of young pigeons^a^.

**Items**	**Control**	**YSE5**	**YSE10**	**YSE15**	***P* value**	**Linear**	**Quadratic**
TP (mg/mL)	26.28 ± 3.11^b^	29.78 ± 3.08^b^	36.31 ± 4.75^c^	30.48 ± 4.97^b^	0.004	0.018	0.011
ALB (g/L)	12.0 ± 2.73	9.22 ± 1.93	9.81 ± 1.12	9.52 ± 2.90	0.169	0.144	0.195
TG (mmol/L)	2.63 ± 0.49	2.34 ± 1.57	2.29 ± 0.49	2.10 ± 0.59	0.758	0.307	0.899
T-CHO (mmol/L)	3.79 ± 0.31^b^	3.69 ± 0.65^bc^	3.05 ± 0.35^d^	3.20 ± 0.34^cd^	0.019	0.007	0.496
T-AOC (mmol/L)	0.94 ± 0.25	0.99 ± 0.27	1.08 ± 0.25	0.97 ± 0.19	0.792	0.686	0.446
T-SOD (U/mL)	19.5 ± 1.5	18.5 ± 1.2	19.2 ± 0.86	19.9 ± 1.6	0.374	0.472	0.158
ALT (U/L)	27.0 ± 16.7^c^	11.4 ± 6.25^b^	8.24 ± 6.67^b^	19.4 ± 9.28^bc^	0.036	0.205	0.008
AST (U/L)	84.4 ± 12.3	89.2 ± 7.40	70.9 ± 16.9	71.4 ± 15.8	0.176	0.069	0.747

a n = 6 replicates (5 young pigeons per replicate) per treatment.

^b−d^Within a row with no common superscripts differ significantly (*P* < 0.05).

Control, 0.9% NaCl solution; YSE5, 5 mg/mL YSE; YSE10, 10 mg/mL YSE; YSE15, 15 mg/mL YSE.

TP, total protein; ALB, albumin; TG, total triglyceride; T-CHO, total cholesterol; ALT, alanine transaminase; AST, aspartate transaminase; T-AOC, total antioxidant capacity; T-SOD, total superoxide dismutase; ALT, alanine aminotransferase; AST, aspartate aminotransferase.

A comparison of serum IgA, IgG, and IgM activity was shown in Table 5. The content of IgM increased significantly linearly with the increase of YSE supplementation (*P* < 0.05), and pigeons gavaged with 10 mg YSE had a higher serum IgM content than the the control pigeons (*P* < 0.05). There was no significant difference in the serum IgG and IgA contents among the four groups (*P* > 0.05).

**Table 5:** Effects of supplemental different levels of YSE on serum immunoglobulins of young pigeons^a^.

**Items**	**Control**	**YSE5**	**YSE10**	**YSE15**	***P* value**	**Linear**	**Quadratic**
IgA (μg/mL)	51.6 ± 17.9	40.3 ± 16.8	43.9 ± 12.6	38.6 ± 17.0	0.532	0.246	0.658
IgG (μg/mL)	312.0 ± 54.5	281.3 ± 50.8	272.2 ± 72.0	297.4 ± 62.8	0.683	0.637	0.272
IgM (μg/mL)	110.7 ± 11.7^b^	106.3 ± 16.1^b^	141.5 ± 7.1^c^	120.4 ± 22.8^b^	0.006	0.040	0.224

a n = 6 replicates (5 young pigeons per replicate) per treatment.

^b, c^Within a row with no common superscripts differ significantly (*P* < 0.05).

Control, 0.9% NaCl solution; YSE5, 5 mg/mL YSE; YSE10, 10 mg/mL YSE; YSE15, 15 mg/mL YSE.

IgA, immunoglobulin A; IgG, immunoglobulin G; IgM, immunoglobulin M.

### Histomorphological parameters in the small intestine

The duodenum, jejunum, and ileum morphology, including VH, CD, and VH/CD were shown in Table 6. In the duodenum, no significant differences were observed in the VH, CD, and VH/CD among groups (*P* > 0.05). The VH in the jejunum increased linearly with the increase of YSE supplementation (*P* < 0.05), and compared with the control group, orally supplemental 10 and 15 mg YSE increased significantly the VH (*P* < 0.05). There were no significant differences in the CD and VH/CD among groups (*P* > 0.05). In the ileum, the CD decreased quadratically the increase of YSE supplementation (*P* < 0.05), and pigeons gavaged with 5 mg and 10 mg YSE had lower CD than the the control pigeons (*P* < 0.05). No significant differences were observed between the VH and VH/CD among the four groups (*P* > 0.05).

**Table 6:** Effect of supplemental different levels of YSE on the histomorphological parameters in the small intestine of young pigeons^a^.

**Items**	**Control**	**YSE5**	**YSE10**	**YSE15**	***P* value**	**Linear**	**Quadratic**	
Duodenum	VH (μm)	542.5 ± 45.1	535.6 ± 47.1	605.9 ± 42.4	572.5 ± 56.2	0.233	0.132	0.590
	CD (μm)	54.3 ± 9.84	57.5 ± 13.6	51.0 ± 4.38	53.6 ± 4.70	0.692	0.627	0.942
	VH/CD	10.3 ± 2.09	10.1 ± 2.44	10.3 ± 2.34	9.93 ± 0.89	0.987	0.826	0.939
Jejunum	VH (μm)	344.0 ± 41.2^b^	409.4 ± 41.9^bc^	432.8 ± 61.0^c^	461.2 ± 56.3^c^	0.011	0.002	0.424
	CD (μm)	48.1 ± 4.58	53.0 ± 10.0	54.5 ± 7.82	55.8 ± 5.28	0.411	0.116	0.594
	VH/CD	6.65 ± 1.06	7.92 ± 1.93	7.15 ± 2.39	7.74 ± 2.18	0.597	0.451	0.653
Ileum	VH (μm)	168.9 ± 40.7	134.8 ± 22.4	130.3 ± 53.1	154.5 ± 20.0	0.350	0.520	0.099
	CD (μm)	41.7 ± 3.98^b^	32.6 ± 9.93^c^	31.2 ± 4.80^c^	37.4 ± 1.90^bc^	0.051	0.249	0.011
	VH/CD	4.21 ± 1.14	3.22 ± 0.43	3.79 ± 0.87	3.97 ± 0.54	0.207	0.916	0.089

a n = 6 replicates (5 young pigeons per replicate) per treatment.

^b, c^Within a row with no common superscripts differ significantly (*P* < 0.05).

Control, 0.9% NaCl solution; YSE5, 5 mg/mL YSE; YSE10, 10 mg/mL YSE; YSE15, 15 mg/mL YSE; VH, villus height; CD, crypt depth; VH/CD, the ratio of villus height to crypt depth.

### Intestinal microflora

According to the results of different levels of YSE on serum biochemical and intestinal morphology-related indicators, pigeons gavaged with 10 mg YSE had significantly higher levels of TP and IgM than other three groups. And, pigeons gavaged with 10 and 15 mg YSE had a lower level of T-CHO and a higher VH in jejunum than the control group. Besides, pigeons gavaged with 5 and 10 mg YSE had lower levels of ALT and CD in ileum than the control group. Thus, based on the beneficial effects of YSE on the serum biochemical parameters, immunoglobulin contents, and intestinal morphology, the level of 10 mg YSE was chosen for further experiment. In addition, the ileum is the longest segment among the three small intestinal segments in young pigeons, so the contents of the ileum were chosen to analyze the microbial diversity and composition.

### Histogram analysis-phylum and genus level

**Figure 1:**
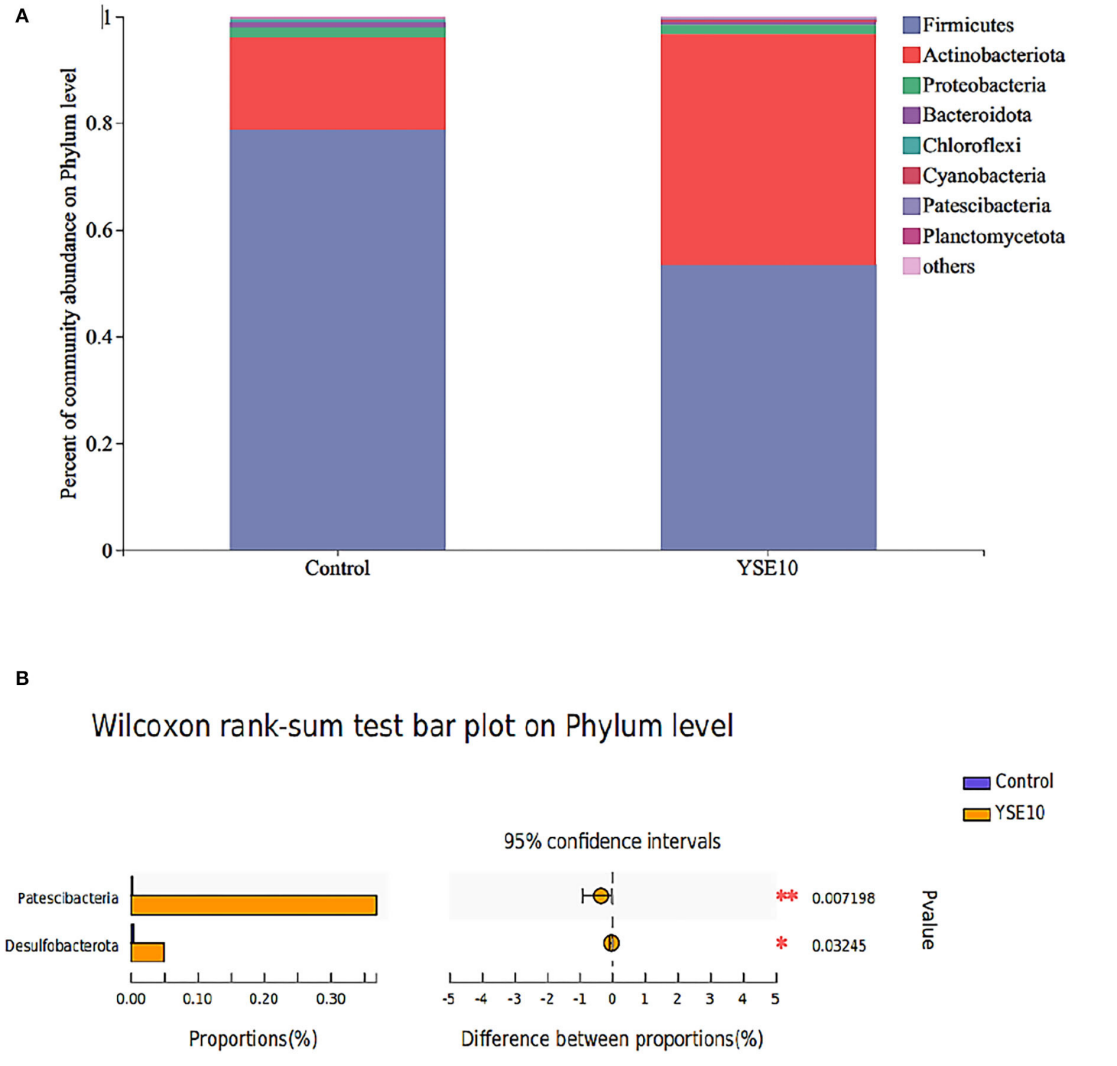
The composition of microbiota and the comparison of differences at the level of phylum in the ileum of young pigeons. **(A)** Percent of community abundance. **(B)** Wilcoxon rank-sum test bar plot. The relative abundances of phylum in all samples' sequence <0.01 are classified as “others”. Control, 0.9% NaCl solution; YSE10, 10 mg/mL YSE.

**Figure 2:**
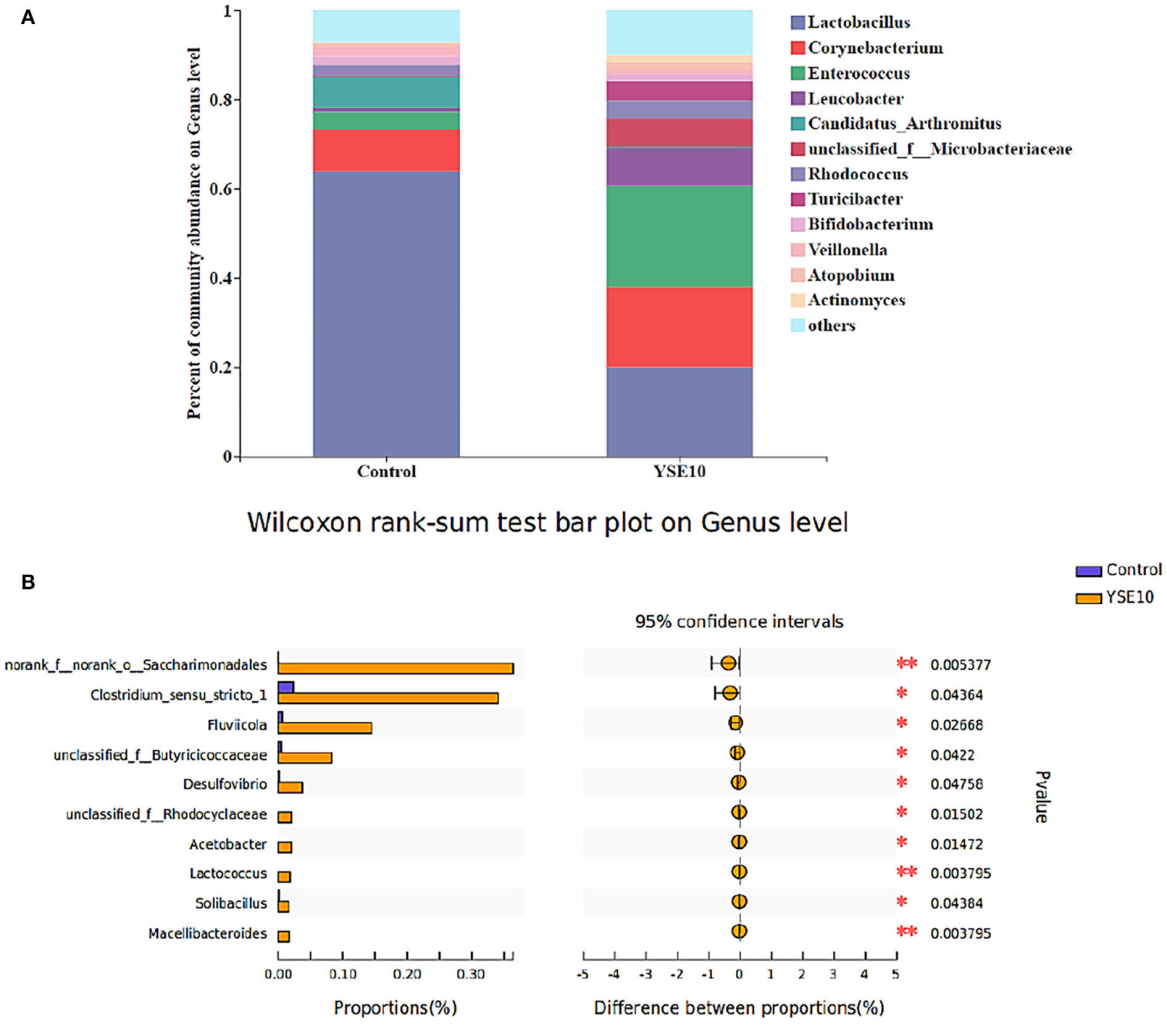
The composition of microbiota and the comparison of differences at the level of genus in the ileum of young pigeons. **(A)** Percent of community abundance. **(B)** Wilcoxon rank-sum test bar plot. The relative abundances of genus in all samples' sequence <0.01 are classified as “others”. Control, 0.9% NaCl solution; YSE10, 10 mg/mL YSE.

As shown in Figure 1A, Firmicutes, Proteobacteria, and Actinobacteria were the main phyla in the ileum, and they accounted for 98.02% in the control group and 98.47% in the YSE10 group. At the phylum level, the analysis results showed that orally supplemental YSE increased the relative abundances of Patescibacteria and Desulfobacterota in the ileum of young pigeons (*P* < 0.05) (Figure 2A).

As shown in Figure 1, the dominant genera of the ileum were *Entrococcus, Leucobacter*, and *unclassified_f_ _Microbacteriaceae*. At the genus level, the analysis results showed that compared with the control group, the relative abundances of *norank_f__norank_o__Saccharimonadales, Clostridium_sensu_stricto_1, unclassified_f__Butyricicoccaceae, Arcobacter, Desulfovibrio, Acetobacter, unclassified_f_ _Rhodocyclaceae, unclassified_f__Carnobacteriaceae, Solibacillus*, and *Lactococcus* were increased in YSE10 group (*P* < 0.05, Figure 2B).

### Alpha diversity of microbial community

**Figure 3:**
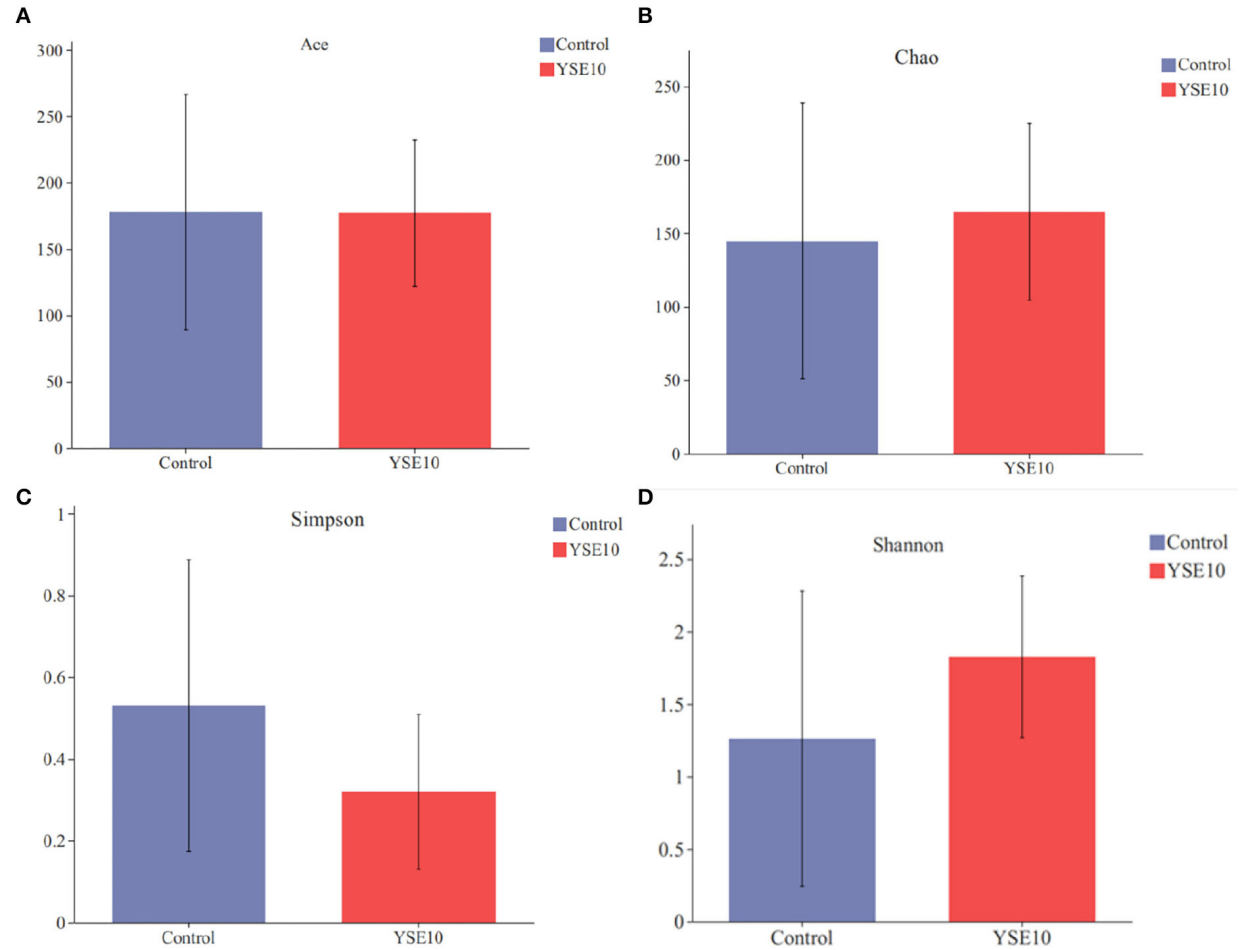
Alpha diversity of two groups. **(A)** Ace index; **(B)** Chao index; **(C)** Simpson index; **(D)** Shannan index. Control, 0.9% NaCl solution; YSE10, 10 mg/mL YSE.

The abundance-based coverage estimator (ACE) index, Chao index, Shannon index, and Simpson index were selected to analyze alpha diversity within a community (Figure 3). There were no significant differences observed among the groups in the ACE index, Chao index, Shannon index, and Simpson index (*P* > 0.05).

### Beta diversity of microbial community

**Figure 4:**
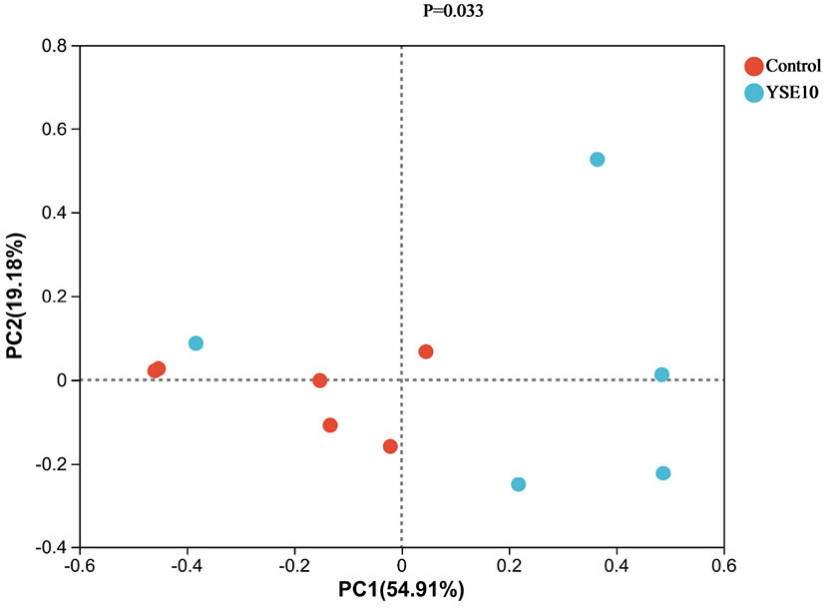
Principal component analysis of operational taxonomic units. PC1, principal coordinate (1) PC2, principal coordinate; (2) Control, 0.9% NaCl solution; YSE10, 10 mg/mL YSE.

Principal co-ordinates analysis (PCoA) was used to analyze the effects of YSE on the beta diversity of the microbial community based on the unweighted UniFrac metric, which was calculated based on the genus and relative abundance of the samples (Figure 4). The result displayed that a significant difference was observed in the diversity of the microbial communities between the control and the YSE10 group (*P* = 0.033).

**Figure 5:**
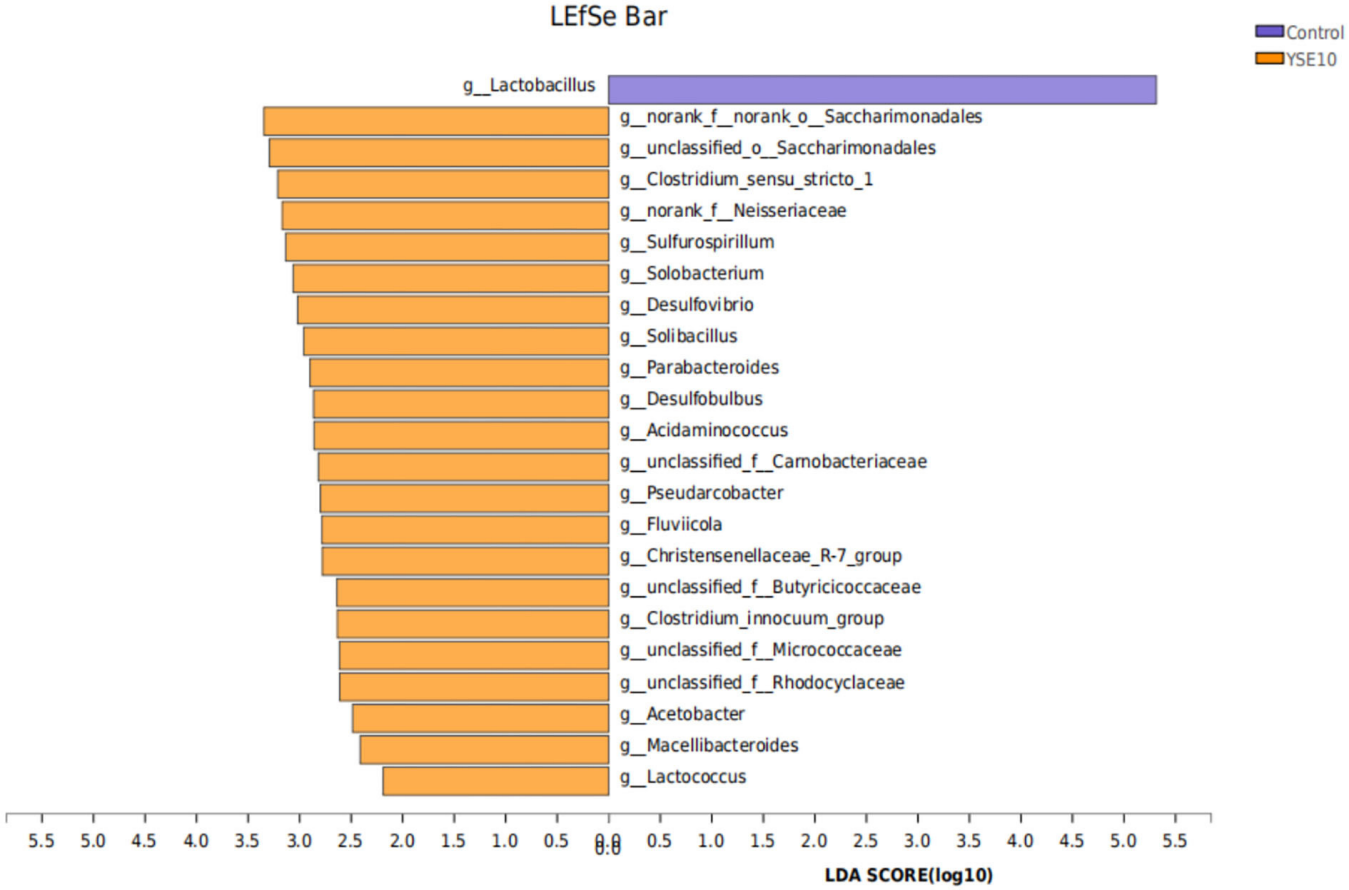
LDA Effect Size (LEfSe) algorithm was used on genus-level OTU tables to determine taxa that best characterize each biological class. Control, 0.9% NaCl solution; YSE10, 10 mg/mL YSE. LDA, linear discriminant analysis.

We used the LDA to determine the taxa that most likely explain the differences between the YSE10 and control samples (Figure 5). Significant differences in microbiota were found when the LEfSe algorithm was used on genus-level OTU tables to determine taxa that characterize each biological class. The result confirmed the bacterial genera *Sulfurospirillum, Solobacterium, Desulfovibrio, Desulfobulbus, Lactococcus, Parabacteroides, Acidaminococcus, Acetobacter*, and *Streptococcus* were significantly enriched in the YSE10 group compared with those in the control.

**Figure 6:**
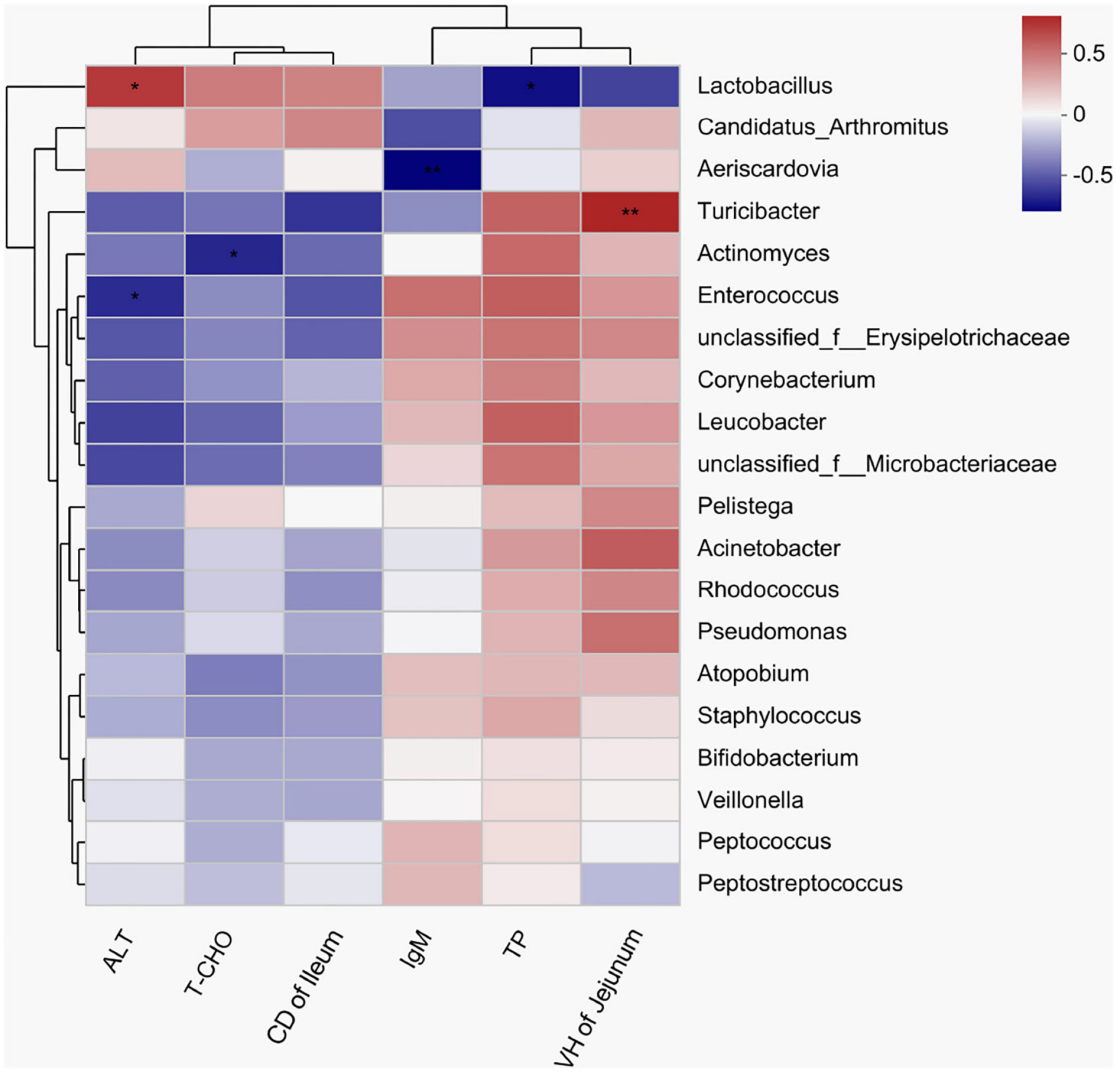
A heatmap showing correlations among the bacterial genera, serum biochemical indexes, and intestinal morphology. *X* and *Y* axes are environmental factors and genera, respectively. Red indicates a positive correlation; blue indicates a negative correlation. *P* < 0.05 is marked with “*”, and *P* < 0.001 is marked with “**”. ALT, alanine aminotransferase; T-CHO, total cholesterol; IgM, immunoglobulin M; TP, total protein.

A heatmap was used to assess the correlations among the bacterial genera, and serum biochemical parameters, immunoglobulin, and intestinal morphological structure by color shades (Figure 6). The correlation heatmap showed that *Enterococcus* had a significantly negative correlation with ALT (*R* = −0.618, *P* = 0.043), but *Lactobacillus* had a significantly positive correlation with ALT (*R* = 0.682, *P* = 0.021). A significantly negative correlation was observed between *Actinomyces* and cholesterol (*R* = −0.633, *P* = 0.036), and a significantly positive correlation was observed between *Turicibacter* and the VH in jejunum (*R* = 0.751, *P* = 0.008).

## Discussion

Different from other poultry, pigeons are fed crop milk and soaked grains by parents in a mouth-to-mouth manner for nearly 28 days after hatching (27, 28). Thereafter, they gradually learn to feed independently, and some pigeons with good performance were reserved as the breeding pigeons. However, the changes of feeding patterns and the separation from breeding parents could bring a “weaning” stress for the 28-day-old young pigeons. And at this time, young pigeons are vulnerable to pathogenic microorganisms such as *Salmonella* and *Trichomonas*, leading to a high mortality (29). Therefore, the young pigeons at 28 days reserved for breeding were focused on in the current study, so as to improve the health condition of young pigeons from the foundation. Previous studies demonstrated that dietary YSE supplementation improved the growth performance of broiler chickens during 1–42 days (30, 31). Unfortunately, the growth performance of young pigeons after adding YSE did not improve significantly in present study, and similar results had been reported in laying quails and hens (20, 32). The body weight of pigeons changed very little after 28 days, implying that the 28-day-old pigeons have reached the mature body weight (33). Perhaps YSE might be more effective in growth performance of the fast-growing broilers or squabs, not in the young pigeons. Besides, it may be caused by low levels of saponins (2.7%) and phenolic compounds (0.8%) in our YSE that could not improve digestion, absorption, and utilization of nutrients in the digestive tract (11).

In addition, the thymus, bursa of Fabricius and spleen are important immune organs of birds, and the development of immune organs has an important impact on the immune function of the body (34). However, in our study, YSE did not result in any discernable effects on the weight of these organs. It is possible that the experiment duration (14 days) is too short to assess the effects of YSE on immune organs. In addition to immune organs, immune molecules also play important roles in regulating immune reactions among the immune system (35). Serum IgG, IgM, and IgA contents are essential immune molecules to measure the functional status of the humoral immune system (36). In our study, the serum IgM level in the YSE10 group was significantly higher than in the control group. Dietary supplemental YSE significantly increased the IgG level observed in laying hens and broilers (11, 37). These results indicated that YSE seems to be more advantageous in production of immunoglobulin. The main task of immunoglobulin in the immune system is to neutralize microorganisms and toxins *via* the nonspecific defense system of body. The steroidal saponin is one of the main chemical constituents of *Y. schidigera* extract, which is most studied and has been shown to be capable of enhancing the natural immune response by stimulating the secretion of cytokines and triggering antibody production and humoral and cellular immune responses (38). Most notably, whether the high level of polysaccharides in our YSE could have functions in regulating immunity needs further demonstration. At present, there are few published studies on the role of polysaccharides from YSE. Therefore, the actual mechanisms involved in the protective effects of YSE remain undefined.

Many serum biochemical indicators can reflect the changes in nutrient metabolism and organ functions of animals (39). Serum glucose, TG and TP contents commonly reflect sugar, fat and protein metabolism in animals (40). In this study, orally supplemental 10 mg YSE increased the serum TP contents, indicating the optimum level of YSE may raise the utilization of TP and effectively inhibit the catabolism of reserve TP in young pigeons. The transaminases of ALT and AST are broadly present in mitochondria and have become the primary screening tool for detecting liver injury (41). The serum ALT level in the YSE10 group was significantly lower than that in the control group. The low level of liver enzymes may be attributable to the repair of hepatic injury in birds administered with the lemongrass essential oil (42). Lemongrass has a cytoprotective effect because of its phenolic components, and fortunately, YSE is also rich in phenolic components (43, 44). We inferred that YSE may have a positive effect on improving liver function. Further investigations are expected to confirm the speculation. The concentration of serum T-CHO was used to evaluate the serum lipid level, which could reflect the health status of the body. Saponins can combine with cholesterol to form water-insoluble complexes, accelerating the conversion rate of cholesterol in the liver to bile acid, and the content of cholesterol in the serum was reduced (45). Previous studies showed that dietary supplemental YSE decreased the serum T-CHO concentration in laying hens and quails (20, 46). In this study, orally supplemental 10 and 15 mg YSE decreased serum T-CHO concentration in young pigeons, which is consistent with previous studies. In addition, it was reported that dietary supplemental YSE could increase the villus height and the villus height/crypt depth ratio of the intestine in broiler chickens (30, 47). Our current study showed that orally supplemental YSE increased the villus height in the jejunum, and reduced the crypt depth in the ileum. It is concluded that YSE may promote intestinal development and enhance the integrity of the intestinal epithelium of young pigeons.

Gut microflora has important effects on the regulation of nutrient utilization, growth performance, energy homeostasis, and immune function by cooperating with the development of the gut system of the host (48, 49). It was reported that *Yucca schidigera* could reduce the diarrhea rate by the modification of the intestinal microbiota in weaned piglets (50). In this study, we found that oral supplementation of YSE enriched the abundance of Patescibacteria and Desulfobacterota phyla. In addition, the genera *Sulfurospirillum, Solobacterium, Desulfovibrio, Desulfobulbus, Lactococcus, Parabacteroides, Acidaminococcus*, and *Acetobacter* were markedly increased in the YSE group. *Desulfococcus* and *Desulfovibrio* are the main components of sulfate-reducing bacteria (SRB), and *Desulfovibrio* may possess a variety of enzymes acting together to resist oxidative stress (51). Meantime, the correlation results showed that *Actinomyces* was negatively correlated with cholesterol, and serum cholesterol in the YSE group decreased. The enzyme obtained from the mycelium of *Actinomyces* lavendula can effectively decrease cholesterol content (52). These results indicated that the YSE may reduce serum cholesterol levels by regulating the abundance of *Actinomyces* genus in the ileum. *Actinobacteria* can produce a wide range of secondary metabolites with structural diversity and are also the source of two-thirds of natural antibiotics and a series of anti-cancer, anti-worm, antifungal and immunosuppressive drugs (53). Moreover, various studies investigated that the *Desulfobacterota* that has been found in the environment, but the proportion in intestinal microorganisms is relatively small and its function is seldom reported (54). In our study, *Enterococcus* was negatively correlated with ALT. Dietary supplementation with *Enterococcus faecium R1* significantly reduced the activity of AST in the blood, so as to attenuate liver injury in LPS-challenged piglets (55). Therefore, our study indicated that the YSE supplementation has important influences on the intestinal microbial composition, and the serum biochemical parameters may alter as the changes in microbial communities.

## Conclusions

In conclusion, orally supplemental 10 mg YSE could enhance the humoral immune function *via* increasing IgM content, regulate the serum biochemical indexes by decreasing the contents of alanine aminotransferase and total cholesterol, and improve intestinal health by promoting intestinal morphology and altering the beta diversity and composition of ileal microbiota. Therefore, YSE can be developed as a feed additive to boost the immunity and intestinal healthy of young pigeons.

## Data availability statement

The datasets presented in this study can be found in online repositories. The names of the repository/repositories and accession number(s) can be found in the article/supplementary material.

## Ethics statement

The animal study was reviewed and approved by Animal Care and Use Committee (Permit number: SYXK-2017-0005) of the Institute of Animal Husbandry and Veterinary Medicine, Beijing Academy of Agriculture and Forestry Sciences (IAHVM-BAAFS), Beijing, China; Animal Care and Use Committee of IAHVM-BAAFS.

## Author contributions

XS drafted the manuscript and conducted the experiment. XL and SD assisted the experiment. ZW and DL conceived the idea. YS modified the manuscript. All authors contributed to the article and approved the submitted version.
